# Clinical and economic outcomes after sternotomy for cardiac surgery with skin closure through 2-octyl cyanoacrylate plus polymer mesh tape versus absorbable sutures plus waterproof wound dressings: a retrospective cohort study

**DOI:** 10.1186/s13019-022-01956-x

**Published:** 2022-08-28

**Authors:** Bob Kiaii, Stephen S. Johnston, Se Ryeong Jang, Nivesh Elangovanraaj, Pranjal Tewari, Brian Po-Han Chen

**Affiliations:** 1 Division of Cardiothoracic Surgery, UC Davis Medical Center, Sacramento, USA; 2 MedTech Epidemiology and Real-World Data Sciences, Johnson & Johnson, 410 George Street, New Brunswick, NJ 08901 USA; 3 Franchise Health Economics and Market Access, Ethicon, Raritan, NJ USA; 4 Mu Sigma, Bangalore, India

**Keywords:** Sternotomy, Skin closure, Absorbable sutures, 2-octyl cyanoacrylate

## Abstract

**Background:**

To compare clinical and economic outcomes after sternotomy for cardiac surgery with skin closure through 2-octyl cyanoacrylate plus polymer mesh tape (2OPMT) versus conventional absorbable sutures plus waterproof wound dressings (CSWWD).

**Methods:**

Retrospective study using the Premier Healthcare Database. Patients undergoing a cardiac surgery requiring sternotomy with 2OPMT or CSWWD were included. Primary outcome was 60-day cumulative incidence of diagnosis for wound complications (infection, dehiscence). Secondary outcomes were index admission hospital length of stay (LOS), total hospital-borne costs, discharge status, and 60-day cumulative incidences of inpatient readmission and reoperation. After propensity score matching, outcomes were compared between the 2OPMT and CSWWD groups using bivariate multilevel mixed-effects generalized linear models.

**Results:**

Overall, 7,901 2OPMT patients and 10,775 CSWWD patients were eligible for study. After propensity score matching on 68 variables, each group comprised 5,338 patients (total study N = 10,676). The 2OPMT and CSWWD groups did not differ significantly in terms of the 60-day cumulative incidences of wound complication (3.47% vs 3.47%, p = 0.996), inpatient readmission (12.6% vs. 13.6%, p = 0.354), and reoperation (10.3% vs 10.1%, p = 0.808), as well as discharge to home versus non-home setting (77.2% vs. 75.1%), p = 0.254. However, the 2OPMT group had significantly lower LOS (9.2 days vs 10.6 days, p < 0.001) and total hospital-borne costs ($50,174 vs $60,526, p < 0.001).

**Conclusions:**

This large observational study provides evidence that sternotomy skin closure with 2OPMT is associated with nearly identical 60-day cumulative incidence of wound complication as compared with CSWWD, while exhibiting a significant association with lower LOS and total hospital-borne costs.

*Trial registration* Not applicable.

**Supplementary Information:**

The online version contains supplementary material available at 10.1186/s13019-022-01956-x.

## Background

Cardiac surgical procedures requiring median sternotomy, such as coronary artery bypass graft, are performed in hundreds of thousands of individuals in the United States and worldwide annually [1, 2]. Successful closure of the sternotomy, including watertight closure at the skin layer, plays a critical role in the prevention of surgical site infection and sternal dehiscence. Sternotomy wound closure typically involves re-approximation of the sternal edges with stainless steel surgical wires followed by subcutaneous suturing; however, closure at the skin layer may be performed via several alternative means, including conventional suturing or sutureless techniques such as topical skin adhesives [3].

Presently, very little published evidence exists regarding the comparative outcomes of conventional suturing versus topical skin adhesives for sternotomy skin closure. Souza et al. reported a statistically significant reduction in the postoperative infection from 4.9 to 2.1% among 1,360 patients after introducing 2-octyl cyanoacrylate as an add-on measure to conventional suturing for sternal skin closure in a single cardiac unit [4]. Fraeman et al. reported that among 59,006 patients undergoing CABG, 2-octyl cyanoacrylate as an add-on measure to conventional suturing for sternal skin closure was associated with a significantly lower rate of surgical site infection as compared with several other types of skin closure [5].

To our knowledge, however, there are no published studies reporting on the comparative outcomes of sternotomy skin closure through conventional sutures versus the skin closure system that combines 2-octyl cyanoacrylate with a self-adhesive polymer mesh tape (DERMABOND® PRINEO® Skin Closure System; Ethicon Inc, NJ, USA; henceforth “2OPMT”; Additional File 5). Several attributes of 2OPMT may influence differential sternotomy skin closure outcomes relative to conventional sutures including antimicrobial protection, strength equivalent to a 3–0 suture and superior to 4–0 suture or staples, optimal distribution of tension, potential for easier self-care (e.g., ability to shower immediately after procedures, no need for dressing changes), and no need for return visits for staple removal [6, 7].

Therefore, we conducted a retrospective study to compare the clinical and economic outcomes of cardiac surgery requiring median sternotomy with skin closure through conventional sutures versus 2OPMT.

## Methods

### Data and patient selection

We conducted this protocol-driven, retrospective study under the exemption from Institutional Review Board oversight for US-based studies using de-identified healthcare records, as dictated by Title 45 Code of Federal Regulations (45 CFR 46.101(b)(4)). Due to the de-identified and retrospective nature of this study, informed consent was not applicable. We extracted the study data from the Premier Healthcare Database® (PHD), which is a population-based hospital research database that contains administrative and electronic health records routinely contributed by over 900 US hospitals that are members of the Premier healthcare performance improvement alliance, representing approximately 25% of annual US inpatient discharges [8]. The PHD database includes discharge-level information on patient demographics, diagnoses, procedures, medical supplies, length of stay, costs, discharge status, and hospital and provider characteristics, among other elements.

We selected patients for study from the PHD if they underwent a cardiac surgery requiring median sternotomy (CAGB, valve repair/replacement, or aortic procedures) during an inpatient admission between October 1, 2015, to June 30, 2020; the first of such admissions observed during this period was defined as the *index admission*. We required patients to be aged 18 years or older at the time of the index admission and to have non-missing data on hospital costs (affecting < 1% of patients). Finally, we included only patients who were admitted to hospitals that continued to contribute data to the PHD for at least 60 days after the patient was discharged from the hospital, which was necessary for measurement of 60-day outcomes as described further below.

### Study groups

Wound closures were performed as per surgeon preference. Using hospital charge master records, which document medical supplies used during admissions, we identified patients for whom skin closure was performed with either 2OPMT or conventional sutures (MONOCRYL® 3–0 or 4–0). Patients with skin closure via conventional sutures were further required to have any use of waterproof wound dressings, which are used in a similar fashion to 2OPMT in terms of creating a waterproof barrier over the surgical incision. We required patients undergoing CABG to have saphenous vein harvesting via only the endoscopic approach to reduce the likelihood of 2OPMT use at the harvesting site versus sternotomy site. We excluded patients in the 2OPMT group if they had use of similar conventional sutures, waterproof wound dressings, or skin staples. We excluded patients in the conventional sutures group if they had use of 2OPMT or skin staples. We identified skin closure devices via combinations of product names and abbreviations (e.g., ‘DERMABOND PRINEO, ‘PRINEO) and/or model numbers (e.g., ‘CLR222US). To maximize the classification accuracy of the skin closure identification algorithms, two separate authors manually reviewed the hospital charge master records. Ultimately, the study comprised two mutually-exclusive skin closure groups: the 2OPMT group and the conventional sutures plus waterproof wound dressings group (CSWWD).

### Measurement of clinical and economic outcomes

The primary study outcome was the cumulative incidence of a composite measure of diagnosed wound complications documented during the index admission or within inpatient, emergency room, or outpatient encounters to the same hospital within 60 days thereafter. The composite measure of wound complications included mediastinitis/abscess, dehiscence at the sternotomy, dehiscence (site unspecified), osteomyelitis, deep sternal wound surgical site infection, and surgical site infection (site unspecified). Diagnoses for wound complications that were designated as Present on Admission were not included for outcome identification during the index admission but were allowed for subsequent hospital encounters.

Economic outcomes included the index admission’s hospital length of stay and total hospital-borne costs (i.e., the costs of the index admission from the hospital’s perspective, inflation-adjusted to 2020 US dollars using the Medical Care component of the US Bureau of Labor Statistics Consumer Price Index), discharge to home (discharge to home with or without home healthcare assistance versus discharge to a skilled nursing facility or other non-home setting), and 60-day cumulative incidences of inpatient readmission and reoperation.

### Measurement of patient and hospital/provider characteristics

We measured the following patient demographics during the index admission: age, sex,, marital status, payer type, and year of index discharge. Patient clinical characteristics, based on diagnosis codes recorded during the index admission for which there was not a specific designation of not being Present on Admission, included: Quan and colleagues’ adaptation of the Charlson Comorbidity Index Score and selected individual comorbidities from the Elixhauser index [9].

Primary diagnosis groupings, created to account for the indication of the cardiac surgery, included: atherosclerotic heart disease of native coronary artery, chronic disease of rheumatic origin, non-ST elevation myocardial infarction, nonrheumatic valve disorder, other circulatory disease. We also created indicators for admission type (elective, emergency, urgent, trauma center, unknown), history of cardiac surgery, aortic procedure, valve repair/replacement procedure, internal mammary artery bypass graft, and number of bypasses (0–5 bypasses) during the index admission.

Hospital/provider characteristics included: urban versus rural hospital, hospital teaching status, hospital geographic region, hospital bed size, hospital annual procedure volume for cardiac surgeries requiring median sternotomy, procedural physician specialty (cardiovascular/thoracic surgeon, internal medicine, other), and an indicator for whether hospital costs are derived from a cost-to-charge ratio versus procedural costing.

### Statistical analyses

We used nearest neighbor propensity score matching to adjust for potential biases which are inherent to observational studies. We matched patients in the 2OPMT group to those in the CSWWD group on all patient and hospital/provider characteristics described above at a 1:1 ratio, without replacement, applying a caliper equal to 0.10 of the standard deviation of the propensity score. We examined the standardized mean differences of covariates after matching to verify appropriate balance, as indicated by an absolute value ≤ 0.10 [10].

In the propensity score matched sample, we used bivariate multilevel mixed-effects generalized linear models to compare the study outcomes between the 2OPMT and CSWWD groups, setting a p-value ≤ 0.05 as the threshold for statistical significance. These models specifically account for hospital level-clustering and the nested patient-within-hospital nature of the study data.

### Post-hoc sensitivity analysis

After observing the results to the analyses outlined in the prospectively-planned protocol, we conducted a post-hoc sensitivity analysis to test the robustness of the findings related to the primary study outcome – 60-day cumulative incidence of wound complication. Specifically, we tested the impact of treating the index admission’s hospital length of stay and total hospital-borne costs as markers of case complexity, as opposed to treating them as outcomes and being in the causal pathway.

## Results

**Fig. 1:**
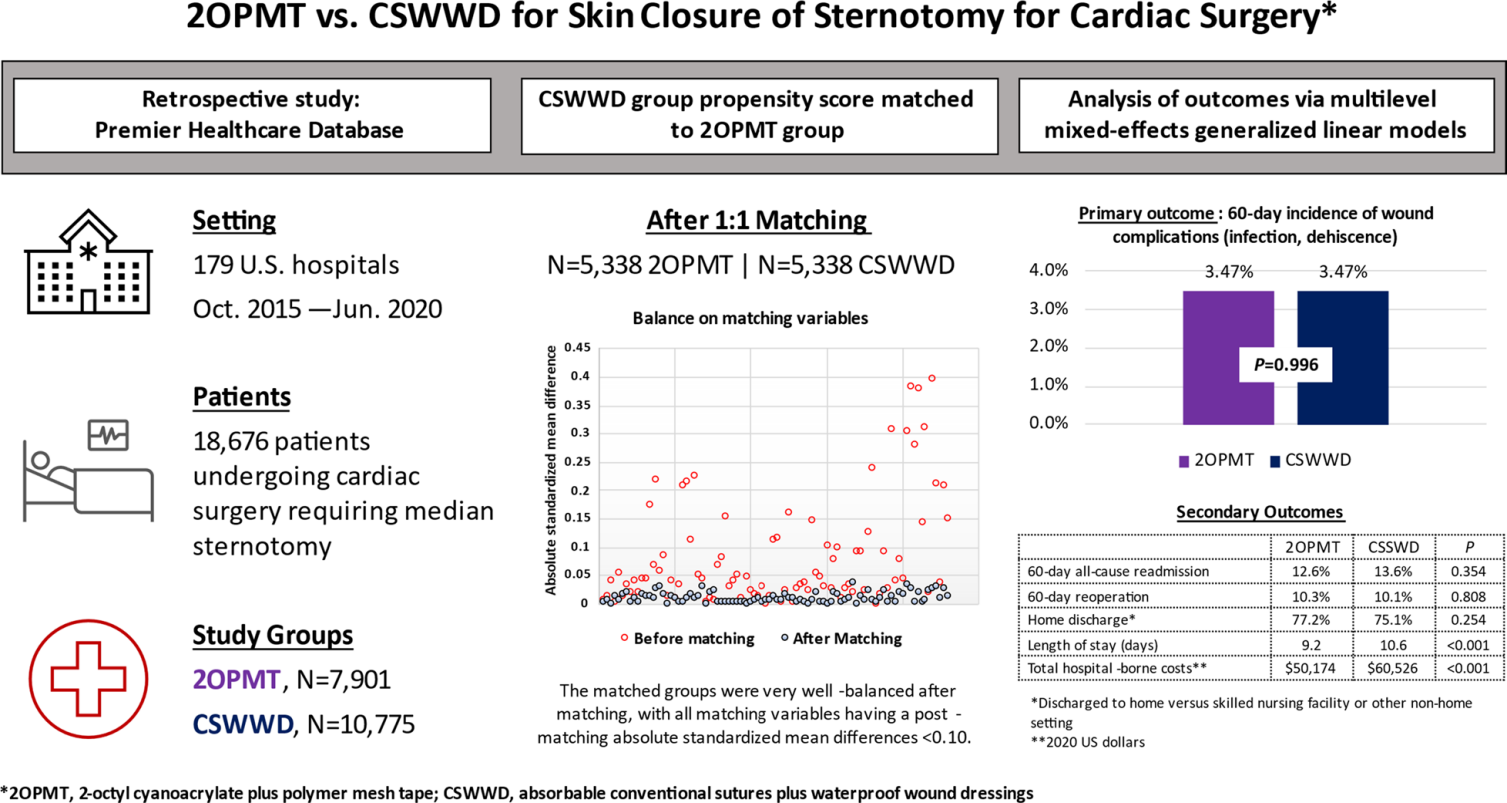
Graphical abstract

Figure 1 presents a graphical abstract of the study’s key design elements and results.

### Propensity score matching

A total of 18,676 patients undergoing cardiac surgery requiring median sternotomy with use of either 2OPMT or CSWWD for skin closure were identified in the database (7,901 in the 2OPMT group; 10,775 in the CSWWD group). Before propensity score matching, the study groups were generally like one another in terms of most patient demographics, clinical characteristics, procedural/admission characteristics, and hospital/provider characteristics (see Additional Files 1, 2, 3, 4).

**Fig. 2:**
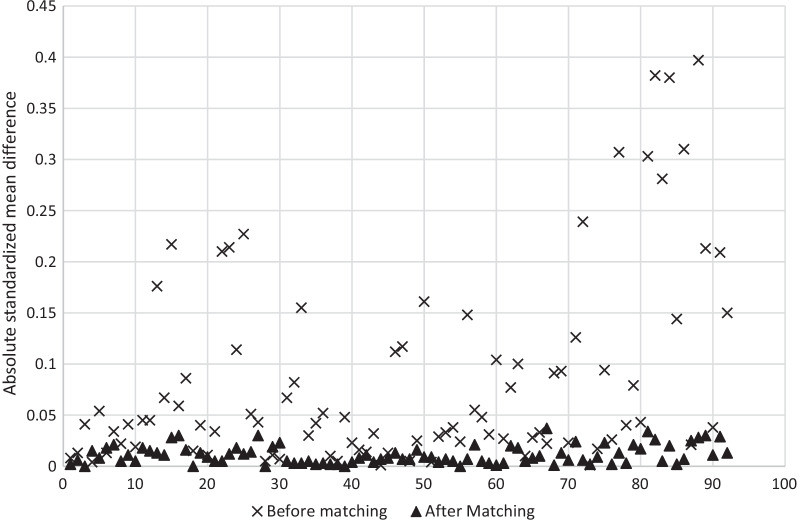
Absolute standardized mean differences for matching variables before and after propensity score matching. *A standardized mean difference with an absolute value ≤ 0.10 is considered to balanced

After propensity score matching, there were 5,338 patients in the 2OPMT group matched to 5,338 patients in the CSWWD group, collectively coming from 137 total hospitals. The matched groups were very well-balanced on matching variables used in the propensity score. Figure 2 shows absolute standardized mean differences for the matching variables before and after propensity score matching. Before propensity score matching, the mean (range) of standardized mean difference values for matching variables was 0.084 (0.001–0.397) versus 0.011 (0.000–0.037) afterwards.

### Patient, procedure/admission, and hospital/provider characteristics

Post-match descriptive statistics on patient demographics, patient clinical characteristics, procedure/admission characteristics, and hospital/provider characteristics are shown in in Tables 1, 2, 3 and 4, respectively. In both groups, the average age of patients was 65 years, most patients were male (72%), white race (84–85%), and most were married (60%). The most common primary diagnoses were related to atherosclerotic heart disease of native coronary artery (50%). Overall, approximately 82% of patients underwent CABG, 25–26% had a valve repair/replacement procedure, and 7% had an aortic procedure. Approximately 75% of patients had internal mammary artery bypass graft..

**Table 1:** Patient demographic characteristics of study groups after propensity score matching

	2OPMT group		CSWWD group		Std. Diff.*
N	5,338	100%	5,338	100%	
Age, mean/SD	65	10.68	65	10.53	0.000
Age category, N/%					
18–34	51	1.00%	52	1.00%	−0.002
35–44	147	2.80%	142	2.70%	0.006
45–54	548	10.30%	548	10.30%	0.000
55–64	1,416	26.50%	1,452	27.20%	−0.015
65–74	2,062	38.60%	2,082	39.00%	−0.008
75–84	1,024	19.20%	986	18.50%	0.018
85+	90	1.70%	76	1.40%	0.021
Female, N/%	1,487	27.90%	1,499	28.10%	−0.005
Marital status, N/%					
Married	3,230	60.50%	3,201	60.00%	0.011
Single	1,864	34.90%	1,877	35.20%	−0.005
Other	229	4.30%	249	4.70%	−0.018
Unknown	15	0.30%	11	0.20%	0.015
Race, N/%					
Black	401	7.50%	419	7.80%	−0.013
Asian	86	1.60%	79	1.50%	0.011
White	4,554	85.30%	4,500	84.30%	0.028
Other	239	4.50%	273	5.10%	−0.030
Unknown	58	1.10%	67	1.30%	−0.016
Payer, N/%					
Commercial	1,393	26.10%	1,392	26.10%	0.000
Medicaid	375	7.00%	393	7.40%	−0.013
Medicare	3,177	59.50%	3,153	59.10%	0.009
Other	393	7.40%	400	7.50%	−0.005
Discharge Year, N/%					
2015 (October onwards)	69	1.30%	72	1.30%	−0.005
2016	693	13.00%	671	12.60%	0.012
2017	1,185	22.20%	1,146	21.50%	0.018
2018	1,331	24.90%	1,304	24.40%	0.012
2019	1,469	27.50%	1,503	28.20%	−0.014
2020 (through June)	591	11.10%	642	12.00%	−0.030

*SD* standard deviation; *Std. Diff.* standardized mean difference

*A standardized mean difference with an absolute value ≤ 0.10 is considered to balanced

**Table 2:** Patient clinical characteristics of study groups after propensity score matching

	2OPMT group		CSWWD group		Std. Diff.*
N	5,338	100%	5,338	100%	
Comorbidities, N/%					
Alcohol abuse	183	3.40%	183	3.40%	0.000
Cancer	45	0.80%	55	1.00%	0.019
Cardiac arrhythmias	1,291	24.20%	1,344	25.20%	−0.023
Chronic pulmonary disease	1,287	24.10%	1,275	23.90%	0.005
Coagulopathy	399	7.50%	395	7.40%	0.003
Congestive heart failure	1,813	34.00%	1,821	34.10%	−0.003
Deficiency anemia	114	2.10%	118	2.20%	−0.005
Depression	549	10.3%	552	10.3%	−0.002
Diabetes, complicated	1,496	28.00%	1,502	28.10%	−0.003
Diabetes, uncomplicated	937	17.60%	933	17.50%	0.002
Drug abuse	174	3.30%	176	3.30%	−0.002
Hypertension, uncomplicated	2,675	50.10%	2,676	50.10%	0.000
Hypertension, complicated	2,123	39.80%	2,113	39.60%	0.004
Hypothyroidism	687	12.90%	673	12.60%	0.008
Liver disease	150	2.80%	160	3.00%	−0.011
Obesity	1,676	31.40%	1,687	31.60%	−0.004
Neurological disorders	162	3.00%	156	2.90%	0.007
Paralysis	13	0.20%	11	0.20%	0.008
Peripheral vascular disease	958	17.90%	985	18.50%	−0.013
Pulmonary circulation disorders	408	7.60%	418	7.80%	−0.007
Renal failure	1,181	22.10%	1,196	22.40%	0.007
RA/collagen vascular diseases	152	2.80%	138	2.60%	0.016
Valvular disease	1,870	35.00%	1,892	35.40%	−0.009
Weight loss	101	1.90%	108	2.00%	−0.009
CCI, N/%					
0	694	13.00%	701	13.10%	−0.004
1–2	2,450	45.90%	2,432	45.60%	0.007
3–4	1,298	24.30%	1,309	24.50%	−0.005
5+	896	16.80%	896	16.80%	0.000

*CCI* Charlson Comorbidity Index; *RA* rheumatoid arthritis; *Std. Diff.* standardized mean difference

*A standardized mean difference with an absolute value ≤ 0.10 is considered to balanced

**Table 3:** Procedural/admission characteristics of study groups after propensity score matching

	2OPMT group		CSWWD group		Std. Diff.*
N	5,338	100%	5,338	100%	
Primary diagnosis group, N/%					
Atherosclerotic heart disease of native coronary artery	2,691	50.40%	2,673	50.10%	0.007
Chronic disease of rheumatic origin	196	3.70%	218	4.10%	−0.021
Non-ST elevation myocardial infarction	1,088	20.40%	1,077	20.20%	0.005
Nonrheumatic valve disorder	807	15.10%	812	15.20%	−0.003
Other Circulatory Disease	556	10.40%	558	10.50%	−0.001
Aortic procedure, N/%	349	6.50%	353	6.60%	−0.003
Valve repair/replacement procedure, N/%	1,333	25.00%	1,379	25.80%	−0.020
Number of bypasses, N/%					
0 (non-CABG procedure)	949	17.80%	986	18.50%	−0.018
1	221	4.10%	226	4.20%	−0.005
2	831	15.60%	847	15.90%	−0.008
3	1,705	31.90%	1,730	32.40%	−0.010
4	1,222	22.90%	1,140	21.40%	0.037
5	410	7.70%	409	7.70%	0.001
Internal mammary artery bypass graft, N/%	4,013	75.20%	3,984	74.60%	0.013
History of cardiac surgery, N/%	1,212	22.70%	1,198	22.40%	0.006
Admission type, N/%					
Elective	2,924	54.80%	2,989	56.00%	−0.024
Emergency	1,246	23.30%	1,233	23.10%	0.006
Information Not Available	40	0.70%	39	0.70%	0.002
Trauma Center	3	0.10%	2	0.00%	0.009
Urgent	1,125	21.10%	1,075	20.10%	0.023

*Std. Diff.* standardized mean difference

*A standardized mean difference with an absolute value ≤ 0.10 is considered to balanced

**Table 4:** Hospital/provider characteristics of study groups after propensity score matching

	2OPMT group		CSWWD group		Std. Diff.*
N	5,338	100%	5,338	100%	
Urban Hospital, N/%	5,174	96.90%	5,176	97.00%	0.002
Teaching Hospital, N/%	3,113	58.30%	3,148	59.00%	0.013
Hospital Bed Size, N/%					
0–299	727	13.60%	733	13.70%	−0.003
300–499	1,562	29.30%	1,512	28.30%	0.021
500+	3,049	57.10%	3,093	57.90%	−0.017
Geographic region, N/%					
Midwest	763	14.30%	827	15.50%	−0.034
Northeast	708	13.30%	662	12.40%	0.026
South	3,322	62.20%	3,336	62.50%	−0.005
West	545	10.20%	513	9.60%	0.020
Provider annual volume, N/%					
0–200	1,502	28.10%	1,506	28.20%	−0.002
201–350	974	18.20%	989	18.50%	−0.007
351–500	992	18.60%	1,045	19.60%	−0.025
500+	1,870	35.00%	1,798	33.70%	0.028
Procedural physician specialty, N/%					
Cardiovascular/thoracic surgery	5,121	95.90%	5,152	96.50%	−0.030
Internal medicine	16	0.30%	13	0.20%	0.011
Other	201	3.80%	173	3.20%	0.029
Cost-to-charge ratio**, N/%	1,384	25.90%	1,353	25.30%	0.013

*SD* standard deviation; *Std. Diff.* standardized mean difference

*A standardized mean difference with an absolute value ≤ 0.10 is considered to balanced

**Hospital costs are derived from a cost-to-charge ratio versus procedural costing

### Propensity score matched analysis of outcomes

Results of the propensity score matched analysis of outcomes are shown in Table 5. The primary study outcome – the cumulative incidence of a composite measure of diagnosed wound complications occurring during the index admission or within 60 days thereafter – occurred at a nearly identical rate between the 2OPMT and CSWWD groups (3.47% 2OPMT, 3.47% CSWWD, *p* = 0.996). There were no statistically significant differences between the 2OPMT and CSWWD groups for any of the constituent parts of the composite measure of diagnosed wound complications (mediastinitis/abscess, dehiscence at the sternotomy, dehiscence [site unspecified], osteomyelitis, deep sternal wound surgical site infection, and surgical site infection [site unspecified]), nor for the cumulative 60-day incidences of all-cause readmission or reoperation.

**Table 5:** Comparison of outcomes in the propensity score matched groups

		2OPMT group	CSWWD group	IDM (95% CI)	p*
		5,338	5,338		
60-day wound complications	Adjusted effects*				
Any wound complication	% (95% CI)	3.47% (2.82–4.12%)	3.47% (2.81–4.13%)	0.00% (−0.91 to 0.91%)	0.996
Mediastinitis/abscess	% (95% CI)	0.17% (0.06–0.28%)	0.11% (0.02–0.20%)	0.06% (−0.09 to 0.20%)	0.438
Sternotomy dehiscence	% (95% CI)	0.67% (0.41–0.93%)	0.89% (0.57–1.20%)	−0.22% (−0.61 to 0.17%)	0.277
Dehiscence (site unspecified)	% (95% CI)	1.46% (1.14–1.78%)	1.42% (1.11–1.74%)	0.04% (−0.42 to 0.49%)	0.872
Osteomyelitis	% (95% CI)	0.15% (0.04–0.25%)	0.15% (0.04–0.27%)	−0.01% (−0.17 to 0.15%)	0.935
Deep sternal wound SSI	% (95% CI)	0.17% (0.03–0.31%)	0.08% (0.00–0.16%)	0.10% (−0.06 to 0.25%)	0.212
SSI (site unspecified)	% (95% CI)	2.16% (1.70–2.62%)	2.04% (1.59–2.48%)	0.12% (−0.52 to 0.76%)	0.707
60-day all-cause readmission	% (95% CI)	12.6% (11.0–14.2%)	13.6% (11.9–15.3%)	−1.1% (−3.3 to 1.2%)	0.354
60-day reoperation	% (95% CI)	10.3% (8.9–11.7%)	10.1% (8.6–11.5%)	0.2% (−1.7 to 2.2%)	0.808
Home discharge**	% (95% CI)	77.2% (74.6–79.8%)	75.1% (72.3–77.9%)	2.1% (−1.5 to 5.7%)	0.254
Length of stay (days)	Mean (95% CI)	9.2 (8.7–9.7)	10.6 (10.0–11.2)	−1.4 (−2.1 to −0.7)	< 0.001
Total hospital-borne costs***	Mean (95% CI)	$50,174 ($46,727–$53,621)	$60,526 ($56,348–$64,705)	−$10,352 (−$13,628 to −$7,077)	< 0.001

*CI* confidence interval; *IDM* incremental difference in means (CSWWD minus 2OPMT); *RR* risk ratio

*Adjusted effect estimates and p-values are based on multilevel mixed-effects generalized linear models accounting for hospital level-clustering and the nested patient-within-hospital nature of the study data

**Discharged to home versus skilled nursing facility or other non-home setting

***2020 US dollars

There was also no statistically significant difference between the 2OPMT and CSWWD groups in the rate of home discharge from the index admission. However, patients in the 2OPMT group had shorter mean length of stay for the index admission (9.2 days 2OPMT, 10.6 days CSWWD, mean incremental difference of 1.4 days [95% confidence interval 0.7 days–2.1 days], *p* < 0.001) and lower mean total hospital-borne costs ($50,174 2OPMT, $60,526 CSWWD, mean incremental difference $10,352 [95% confidence interval $7,077–$13,628], p < 0.001).

### Post-hoc sensitivity analysis

The post-hoc sensitivity analysis treating the index admission’s hospital length of stay and total hospital-borne costs as covariates within the statistical model that was used to compare the primary outcome between 2OPMT and CSWWD yielded results that were highly consistent with the primary analysis: there was no statistically significant difference in the 60-day cumulative incidence of wound complication between the 2OPMT and CSWWD groups (3.63% 2OPMT, 3.31% CSWWD, *p* = 0.456). Within that model, each additional day of length of stay was associated with a 0.14% increase in the 60-day cumulative incidence of wound complication (*p* < 0.001); however, index admission’s total hospital-borne costs were not significantly associated with the 60-day cumulative incidence of wound complication (*p* = 0.075).

## Discussion

This is the first study to compare clinical and economic outcomes between 2OPMT and CSWWD for sternotomy skin closure among patients undergoing cardiac surgery in usual clinical practice. Among 10,676 patients from 137 US hospitals, sternotomy skin closure with 2OPMT was associated with nearly identical rates of wound complications and potential economic benefits, when compared with CSWWD.

As noted in the Introduction section, there are few other studies to which we can compare the present results. Whereas the present study specifically compared 2OPMT with CSWWD, both Souza et al. and Fraeman et al. reported only on the use of 2-octyl cyanoacrylate component of 2OPMT and both specifically examined it as an add-on strategy to conventional sutures, finding clinical benefits associated with 2-octyl cyanoacrylate [4, 5]. In the present study, we observed a 60-day cumulative incidence of any wound complication, driven largely by dehiscence (site unspecified, 1.46%) and SSI (site unspecified, 2.16%). Overall, the rates of infection in the present study fall within the range of prior studies reported for 2-octyl cyanoacrylate above. Across-study differences in the case mix of patients, follow-up durations, and methods of wound complication ascertainment potentially explain any differences in observed risks.

Sternotomy wound complications are a major cause of morbidity, economic burden, and can sometimes be fatal [11–16]. Accordingly, the Centers for Medicare & Medicaid Services have employed multiple measures to penalize hospitals resulting in mediastinitis (a form of deep sternal wound infection [DSWI]) or high readmission rates for CABG, putting providers under pressure to control the risk of DSWI [17, 18]. The nearly identical rates of wound complications in the present study’s groups provide reassurance that 2OPMT is associated with a clinical profile that is comparable to the more common skin closure technique employing conventional absorbable sutures. These findings related to the primary endpoint were also robust to a conservative post-hoc sensitivity analysis.

Our study adds to a growing body of evidence regarding the association of 2OPMT with beneficial economic and clinical outcomes. Three prior multi-hospital retrospective database studies of 2OPMT in non-cardiac surgeries have reported results suggesting that 2OPMT is associated with similar or better outcomes as compared with other skin closure techniques, specifically focusing on skin staples in the setting of total knee replacement, cesarean section, and spinal fusion surgery [19–21]. Notably, in congruence with the present study, all three prior studies reported that 2OPMT was associated with statistically significant shorter hospital length of stay, 5.4% shorter among patients undergoing cesarean section, 12.5% shorter among patients undergoing total knee replacement, and 13.2% shorter among patients undergoing spinal fusion surgery. A potential driver of the relatively shorter length of stay is that 2OPMT may promote quicker transition to a home setting, given that it allows patients to shower immediately after procedures and obviates the need for wound dressing changes in the hospital.

We conducted additional of post-hoc analyses to further investigate the difference in length of stay between 2OPMT and CSWWD despite a nearly identical 60-day cumulative incidence of any wound complication. First, we examined the association between wound complications that developed during the index admission and the index admission’s length of stay – it is important to note that only the wound complications that occur during the index admission have the potential to influence its length of stay (i.e., those occurring after discharge, which accounted for 70% of wound complications overall, cannot influence length of stay). As expected, we found that wound complications that develop during the index admission are indeed associated with higher length of stay: average length of stay was 9.5 versus 25.9 days among patients without versus with wound complications during the index admission (p < 0.001). Second, we tested the hypothesis that even when adjusting for wound complications that develop during the index admission, 2OPMT will still be significantly associated with lower length of stay: in this analysis, average length of stay was 9.1 days for 2OPMT versus 10.3 for CSWWD (p < 0.001), a difference of 1.2 days as compared with 1.4 days in the primary analysis. The slightly lower difference of 1.2 days (versus 1.4 days) may be explained by the fact that 2OPMT had a lower (though not reaching statistical significance) rate of wound complications that developed during the index admission 0.99% for 2OPMT versus 1.25% for CSWWD (p = 0.4).

Furthermore, 2OPMT was not associated with increased total hospital-borne costs, but rather statistically significant lower costs, as compared with CSWWD. It is important to note that lower costs observed among the 2OPMT group are unlikely to be attributable to use of 2OPMT alone and may have been driven by other aspects of hospitals’ clinical or financial practices. To further investigate this, we examined whether the shorter length of stay for 2OPMT was a substantive driver of the overall cost difference. Specifically, we fit a model in which total hospital cost was the dependent variable and 2OPMT as well as length of stay were the independent variables. In this model, each additional day of length of stay was associated with a 4.7% increase in total hospital costs (p < 0.001), whereas 2OPMT was associated with lower costs ($54,698 for 2OPMT versus $57,409 for CSWWD, p = 0.012). Adjusting for length of stay, therefore, decreased the incremental cost difference from the primary analysis ($10,352) to $2,710 – the difference in cost if the 2OPMT and CSWWD groups had the same duration of length of stay. This finding suggests that the lower length of stay associated with 2OPMT accounts for $7,642 of the observed difference in costs.

### Limitations

This study was subject to limitations. First, the Premier Healthcare Database does not have information on cosmesis outcomes, which can be very important from the patient perspective. Prior studies in the setting of total knee replacement and abdominoplasty have found that 2OPMT was associated with favorable cosmesis outcomes from the patient and physician perspective as compared with other methods of skin closure [22, 23]. Second, this study was retrospective and observational in nature, and therefore causality cannot be established for the observed relationships. To address this, we used propensity score matching on over 92 unique matching variables, resulting in a very well-balanced sample. However, we cannot rule out the possibility of residual confounding from variables that were not available within the database. Third, we relied on ICD-10-CM diagnosis coding to measure wound complications. The use of such codes may lead to measurement error when ascertaining infection and wound complications; however, it is unlikely that measurement error would have systematically differed between the 2OPMT and CSWWD groups. Finally, we relied on hospital charge master records which documented the skin closure supplies used during the cardiac surgeries. These records are unstructured, require manual review of text descriptions for device identification, and may be associated with measurement errors.

## Conclusions

This large observational study using propensity score matching is the first of its kind and provides evidence that sternotomy skin closure with 2OPMT is associated with nearly identical 60-day cumulative incidence of wound complication as compared with CSWWD. In analysis of secondary outcomes, 2OPMT was also associated with similar 60-day cumulative incidences of inpatient readmission and reoperation, as well as discharge to the home versus non home setting as compared with CSWWD, while exhibiting a significant association with lower length of hospital stay and lower total hospital-borne costs.

## Supplementary Information

**Additional file 1. Appendix Table 1**. Patient demographic characteristics of study groups before propensity score matching.

**Additional file 2. Appendix Table 2**. Patient clinical characteristics of study groups before propensity score matching.

**Additional file 3. Appendix Table 3**. Procedural/admission characteristics of study groups before propensity score matching.

**Additional file 4. Appendix Table 4**. Hospital/provider characteristics of study groups before propensity score matching.

**Additional file 5. Appendix Figure 1**. Picture: 2-octyl cyanoacrylate plus polymer mesh tape applied to a sternal wound for skin closure.

## Data Availability

The Premier Healthcare Database® that support the findings of this study are available from Premier Applied Sciences, Premier Inc, Charlotte, North Carolina, but restrictions apply to the availability of these data, which were used under license for the current study, and so are not publicly available. Data are however available from the authors upon reasonable request and with permission of Premier Inc.

## Abbreviations

2OPMT: 2-Octyl cyanoacrylate plus polymer mesh tape
CCI: Charlson Comorbidity Index
CI: Confidence interval
CSWWD: Conventional absorbable sutures plus waterproof wound dressings
ICD-10-CM: International Classification of Diseases, 10th Revision, Clinical Modification
ICD-10-PCS: International Classification of Diseases, 10th Revision, Procedure Classification System
IDM: Incremental difference in means
LOS: Length of stay
PHD: Premier Healthcare Database
RA: Rheumatoid arthritis
RR: Risk ratio
SD: Standard deviation
Std. Diff.: Standardized mean difference
