# Supplementary material for: Clinical and economic outcomes after sternotomy for cardiac surgery with skin closure through 2-octyl cyanoacrylate plus polymer mesh tape versus absorbable sutures plus waterproof wound dressings: a retrospective cohort study
Source: J Cardiothorac Surg. 2022 Aug 28;17:212. doi: 10.1186/s13019-022-01956-x (PMC9420285; doi:10.1186/s13019-022-01956-x)
Supplement: Supplementary file 1 — Additional file 1. Appendix Table 1. Patient demographic characteristics of study groups before propensity score matching. [file 13019_2022_1956_MOESM1_ESM.docx]

Appendix Table 1. Patient demographic characteristics of study groups before propensity score matching

*Each x/diamond represents a standardized difference value corresponding to a single covariate before/after propensity score matching; x’s represent pre-match standardized differences, diamonds represent post-match standardized differences

|  | 2OPMT group | | CSWWD group | | Std.  Diff.* |
| --- | --- | --- | --- | --- | --- |
|  |  | |  | |  |
| N | 7,901 | 100.00% | 10,775 | 100.00% |  |
| Age, mean / SD | 65 | 10.35 | 65 | 10.72 | 0.000 |
| Age category, N / % |  |  |  |  |  |
| 18-34 | 58 | 0.70% | 87 | 0.80% | -0.008 |
| 35-44 | 196 | 2.50% | 289 | 2.70% | -0.013 |
| 45-54 | 774 | 9.80% | 1,190 | 11.00% | -0.041 |
| 55-64 | 2,126 | 26.90% | 2,917 | 27.10% | -0.004 |
| 65-74 | 3,128 | 39.60% | 3,983 | 37.00% | 0.054 |
| 75-84 | 1,500 | 19.00% | 2,099 | 19.50% | -0.013 |
| 85+ | 119 | 1.50% | 210 | 1.90% | -0.034 |
| Female, N / % | 2,163 | 27.40% | 3,055 | 28.40% | -0.022 |
| Marital status, N / % |  |  |  |  |  |
| Married | 4,859 | 61.50% | 6,409 | 59.50% | 0.041 |
| Single | 2,763 | 35.00% | 3,865 | 35.90% | -0.019 |
| Other | 264 | 3.30% | 453 | 4.20% | -0.045 |
| Unknown | 15 | 0.20% | 48 | 0.40% | -0.045 |
| Race, N/% |  |  |  |  |  |
| Black | 443 | 5.60% | 1,117 | 10.40% | -0.176 |
| Asian | 96 | 1.20% | 222 | 2.10% | -0.067 |
| White | 6,988 | 88.40% | 8,688 | 80.60% | 0.217 |
| Other | 310 | 3.90% | 556 | 5.20% | -0.059 |
| Unknown | 64 | 0.80% | 192 | 1.80% | -0.086 |
| Payer, N / % |  |  |  |  |  |
| Commercial | 2,055 | 26.00% | 2,872 | 26.70% | -0.015 |
| Medicaid | 594 | 7.50% | 701 | 6.50% | 0.04 |
| Medicare | 4,663 | 59.00% | 6,299 | 58.50% | 0.011 |
| Other | 589 | 7.50% | 903 | 8.40% | -0.034 |
| Discharge Year, N / % |  |  |  |  |  |
| 2015 (October onwards) | 70 | 0.90% | 448 | 4.20% | -0.21 |
| 2016 | 879 | 11.10% | 2,014 | 18.70% | -0.214 |
| 2017 | 1,904 | 24.10% | 2,092 | 19.40% | 0.114 |
| 2018 | 2,315 | 29.30% | 2,111 | 19.60% | 0.227 |
| 2019 | 1,962 | 24.80% | 2,918 | 27.10% | -0.051 |
| 2020 (through June) | 771 | 9.80% | 1,192 | 11.10% | -0.043 |

*SD, standard deviation; Std. Diff., standardized mean difference*

* A standardized mean difference with an absolute value ≤0.10 is considered to balanced
