# Supplementary material for: Clinical and economic outcomes after sternotomy for cardiac surgery with skin closure through 2-octyl cyanoacrylate plus polymer mesh tape versus absorbable sutures plus waterproof wound dressings: a retrospective cohort study
Source: J Cardiothorac Surg. 2022 Aug 28;17:212. doi: 10.1186/s13019-022-01956-x (PMC9420285; doi:10.1186/s13019-022-01956-x)
Supplement: Supplementary file 2 — Additional file 2. Appendix Table 2. Patient clinical characteristics of study groups before propensity score matching. [file 13019_2022_1956_MOESM2_ESM.docx]

Appendix Table 2. Patient clinical characteristics of study groups before propensity score matching

|  | 2OPMT group | | CSWWD group | | Std.  Diff.* |
| --- | --- | --- | --- | --- | --- |
|  |  | |  | |  |
| N | 7,901 | 100.00% | 10,775 | 100.00% |  |
| Comorbidities, N / % |  |  |  |  |  |
| Alcohol abuse | 266 | 3.40% | 353 | 3.30% | 0.005 |
| Cancer | 127 | 1.20% | 84 | 1.10% | 0.011 |
| Cardiac arrhythmias | 1,950 | 24.70% | 2,690 | 25.00% | -0.007 |
| Chronic pulmonary disease | 1,948 | 24.70% | 2,353 | 21.80% | 0.067 |
| Coagulopathy | 514 | 6.50% | 935 | 8.70% | -0.082 |
| Congestive heart failure | 2,438 | 30.90% | 4,114 | 38.20% | -0.155 |
| Deficiency anemia | 159 | 2.00% | 265 | 2.50% | -0.030 |
| Depression | 831 | 10.5% | 998 | 9.3% | 0.042 |
| Diabetes, complicated | 2,306 | 29.20% | 2,893 | 26.80% | 0.052 |
| Diabetes, uncomplicated | 1,422 | 18.00% | 1,897 | 17.60% | 0.010 |
| Drug abuse | 235 | 3.00% | 311 | 2.90% | 0.005 |
| Hypertension, uncomplicated | 4,040 | 51.10% | 5,251 | 48.70% | 0.048 |
| Hypertension, complicated | 3,102 | 39.30% | 4,352 | 40.40% | -0.023 |
| Hypothyroidism | 1,029 | 13.00% | 1,345 | 12.50% | 0.016 |
| Liver disease | 230 | 2.90% | 339 | 3.10% | -0.014 |
| Obesity | 2,495 | 31.60% | 3,245 | 30.10% | 0.032 |
| Neurological disorders | 227 | 2.90% | 307 | 2.80% | 0.001 |
| Paralysis | 35 | 0.30% | 20 | 0.30% | 0.013 |
| Peripheral vascular disease | 1,296 | 16.40% | 2,237 | 20.80% | -0.112 |
| Pulmonary circulation disorders | 527 | 6.70% | 1,065 | 9.90% | -0.117 |
| Renal disease | 2,482 | 23.00% | 1,804 | 22.80% | 0.005 |
| RA / collagen vascular diseases | 223 | 2.80% | 261 | 2.40% | 0.025 |
| Valvular disease | 2,535 | 32.10% | 4,288 | 39.80% | -0.161 |
| Weight loss | 151 | 1.90% | 200 | 1.90% | 0.004 |
| CCI, N / % |  |  |  |  |  |
| 0 | 1,040 | 13.20% | 1,316 | 12.20% | 0.029 |
| 1-2 | 3,653 | 46.20% | 4,807 | 44.60% | 0.033 |
| 3-4 | 1,887 | 23.90% | 2,751 | 25.50% | -0.038 |
| 5+ | 1,321 | 16.70% | 1,901 | 17.60% | -0.024 |

*CCI, Charlson Comorbidity Index; RA, rheumatoid arthritis; Std. Diff., standardized mean difference*

* A standardized mean difference with an absolute value ≤0.10 is considered to balanced
