# Supplementary material for: Clinical and economic outcomes after sternotomy for cardiac surgery with skin closure through 2-octyl cyanoacrylate plus polymer mesh tape versus absorbable sutures plus waterproof wound dressings: a retrospective cohort study
Source: J Cardiothorac Surg. 2022 Aug 28;17:212. doi: 10.1186/s13019-022-01956-x (PMC9420285; doi:10.1186/s13019-022-01956-x)
Supplement: Supplementary file 3 — Additional file 3. Appendix Table 3. Procedural/admission characteristics of study groups before propensity score matching. [file 13019_2022_1956_MOESM3_ESM.docx]

Appendix Table 3. Procedural/admission characteristics of study groups before propensity score matching

|  | 2OPMT group | | CSWWD group | | Std.  Diff.* |
| --- | --- | --- | --- | --- | --- |
|  |  | |  | |  |
| N | 7,901 | 100.00% | 10,775 | 100.00% |  |
| Primary diagnosis group, N / % |  |  |  |  |  |
| Atherosclerotic heart disease of native coronary artery | 4,176 | 52.90% | 4,898 | 45.50% | 0.148 |
| Chronic disease of rheumatic origin | 280 | 3.50% | 500 | 4.60% | -0.055 |
| Non-ST elevation myocardial infarction | 1,575 | 19.90% | 2,359 | 21.90% | -0.048 |
| Nonrheumatic valve disorder | 1,131 | 14.30% | 1,662 | 15.40% | -0.031 |
| Other Circulatory Disease | 739 | 9.40% | 1,356 | 12.60% | -0.104 |
| Aortic procedure, N / % | 504 | 6.40% | 759 | 7.00% | -0.027 |
| Valve repair/replacement procedure, N / % | 1,864 | 23.60% | 2,904 | 27.00% | -0.077 |
| Number of bypasses, N / % |  |  |  |  |  |
| 0 (non-CABG procedure) | 1,273 | 16.10% | 2,150 | 20.00% | -0.100 |
| 1 | 323 | 4.10% | 462 | 4.30% | -0.010 |
| 2 | 1,300 | 16.50% | 1,664 | 15.40% | 0.028 |
| 3 | 2,601 | 32.90% | 3,380 | 31.40% | 0.033 |
| 4 | 1,726 | 21.80% | 2,451 | 22.70% | -0.022 |
| 5 | 678 | 8.60% | 668 | 6.20% | 0.091 |
| Internal mammary artery bypass graft, N / % | 6,103 | 77.20% | 7,890 | 73.20% | 0.093 |
| History of cardiac surgery, N / % | 1,761 | 22.30% | 2,299 | 21.30% | 0.023 |
| Admission type, N / % |  |  |  |  |  |
| Elective | 4,543 | 57.50% | 5,519 | 51.20% | 0.126 |
| Emergency | 1,503 | 19.00% | 3,142 | 29.20% | -0.239 |
| Information Not Available | 47 | 0.60% | 65 | 0.60% | -0.001 |
| Trauma Center | 4 | 0.10% | 2 | 0.00% | 0.017 |
| Urgent | 1,804 | 22.80% | 2,047 | 19.00% | 0.094 |

*Std. Diff., standardized mean difference*

* A standardized mean difference with an absolute value ≤0.10 is considered to balanced
