# Supplementary material for: Clinical and economic outcomes after sternotomy for cardiac surgery with skin closure through 2-octyl cyanoacrylate plus polymer mesh tape versus absorbable sutures plus waterproof wound dressings: a retrospective cohort study
Source: J Cardiothorac Surg. 2022 Aug 28;17:212. doi: 10.1186/s13019-022-01956-x (PMC9420285; doi:10.1186/s13019-022-01956-x)
Supplement: Supplementary file 4 — Additional file 4. Appendix Table 4. Hospital/provider characteristics of study groups before propensity score matching. [file 13019_2022_1956_MOESM4_ESM.docx]

Appendix Table 4. Hospital/provider characteristics of study groups before propensity score matching

|  | 2OPMT group | | CSWWD group | | Std.  Diff.* |
| --- | --- | --- | --- | --- | --- |
|  |  | |  | |  |
| N | 7,901 | 100.00% | 10,775 | 100.00% |  |
| Urban Hospital, N / % | 7,527 | 95.30% | 10,322 | 95.80% | 0.026 |
| Teaching Hospital, N / % | 4,862 | 61.50% | 5,003 | 46.40% | -0.307 |
| Hospital Bed Size, N / % |  |  |  |  |  |
| 000-299 | 1,428 | 18.10% | 1,783 | 16.50% | 0.040 |
| 300-499 | 2,278 | 28.80% | 3,500 | 32.50% | -0.079 |
| 500+ | 4,195 | 53.10% | 5,492 | 51.00% | 0.043 |
| Geographic region, N / % |  |  |  |  |  |
| Midwest | 825 | 10.40% | 2,307 | 21.40% | -0.303 |
| Northeast | 1,547 | 19.60% | 740 | 6.90% | 0.382 |
| South | 4,146 | 52.50% | 7,126 | 66.10% | -0.281 |
| West | 1,383 | 17.50% | 602 | 5.60% | 0.380 |
| Provider annual volume, N / % |  |  |  |  |  |
| 0-200 | 1,950 | 24.70% | 3,354 | 31.10% | -0.144 |
| 201-350 | 1,129 | 14.30% | 2,870 | 26.60% | -0.310 |
| 351-500 | 1,705 | 21.60% | 2,231 | 20.70% | 0.021 |
| 500+ | 3,117 | 39.50% | 2,320 | 21.50% | 0.397 |
| Procedural physician specialty, N / % |  |  |  |  |  |
| Cardiovascular/thoracic surgery | 7,426 | 94.00% | 10,571 | 98.10% | -0.213 |
| Internal medicine | 23 | 0.30% | 13 | 0.10% | 0.038 |
| Other | 452 | 5.70% | 191 | 1.80% | 0.209 |
| Cost-to-charge ratio**, N / % | 2,082 | 26.40% | 2,157 | 20.00% | 0.150 |

*SD, standard deviation; Std. Diff., standardized mean difference*

* A standardized mean difference with an absolute value ≤0.10 is considered to balanced

** Hospital costs are derived from a cost-to-charge ratio vs. procedural costing
