# Supplementary material for: Clinical and economic outcomes after sternotomy for cardiac surgery with skin closure through 2-octyl cyanoacrylate plus polymer mesh tape versus absorbable sutures plus waterproof wound dressings: a retrospective cohort study
Source: J Cardiothorac Surg. 2022 Aug 28;17:212. doi: 10.1186/s13019-022-01956-x (PMC9420285; doi:10.1186/s13019-022-01956-x)

Appendix Figure 1

Picture Legend: 2-octyl cyanoacrylate plus polymer mesh tape applied to a sternal wound for skin closure


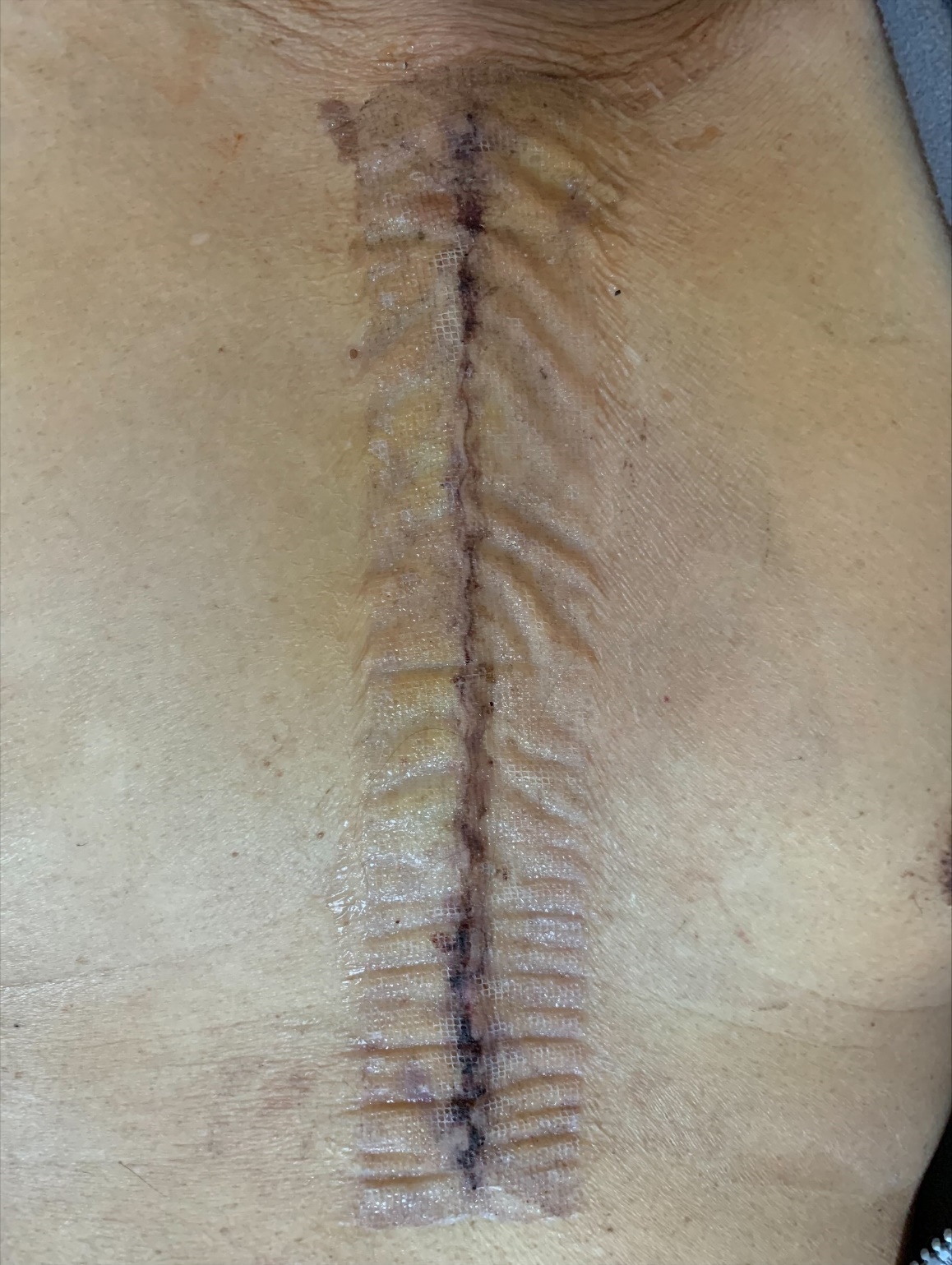

Supplement: Supplementary file 5 — Additional file 5. Appendix Figure 1. Picture: 2-octyl cyanoacrylate plus polymer mesh tape applied to a sternal wound for skin closure. [file 13019_2022_1956_MOESM5_ESM.docx]
